# Voluntary Climate Change Mitigation Actions of Young Adults: A Classification of Mitigators through Latent Class Analysis

**DOI:** 10.1371/journal.pone.0102072

**Published:** 2014-07-23

**Authors:** Essi A. E. Korkala, Timo T. Hugg, Jouni J. K. Jaakkola

**Affiliations:** 1 Center for Environmental and Respiratory Health Research, University of Oulu, Oulu, Finland; 2 Medical Research Center Oulu, Oulu University Hospital and University of Oulu, Oulu, Finland; 3 Public Health, Institute of Health Sciences, University of Oulu, Oulu, Finland; Queensland University of Technology, Australia

## Abstract

Encouraging individuals to take action is important for the overall success of climate change mitigation. Campaigns promoting climate change mitigation could address particular groups of the population on the basis of what kind of mitigation actions the group is already taking. To increase the knowledge of such groups performing similar mitigation actions we conducted a population-based cross-sectional study in Finland. The study population comprised 1623 young adults who returned a self-administered questionnaire (response rate 64%). Our aims were to identify groups of people engaged in similar climate change mitigation actions and to study the gender differences in the grouping. We also determined if socio-demographic characteristics can predict group membership. We performed latent class analysis using 14 mitigation actions as manifest variables. Three classes were identified among men: the Inactive (26%), the Semi-active (63%) and the Active (11%) and two classes among women: the Semi-active (72%) and the Active (28%). The Active among both genders were likely to have mitigated climate change through several actions, such as recycling, using environmentally friendly products, preferring public transport, and conserving energy. The Semi-Active had most probably recycled and preferred public transport because of climate change. The Inactive, a class identified among men only, had very probably done nothing to mitigate climate change. Among males, being single or divorced predicted little involvement in climate change mitigation. Among females, those without tertiary degree and those with annual income €≥16801 were less involved in climate change mitigation. Our results illustrate to what extent young adults are engaged in climate change mitigation, which factors predict little involvement in mitigation and give insight to which segments of the public could be the audiences of targeted mitigation campaigns.

## Introduction

Encouraging individuals to take action is important for the overall success of climate change mitigation. This is because individuals’ lifestyles and consumption patterns have influence on several greenhouse gas producing sectors such as the energy industries, transportation and residential sector. For example in the European Union these sectors together accounted for more than 70% of the total greenhouse gas emission in 2007 [1].

The role of individuals in climate change mitigation is small when compared to the roles of national governments or international policymakers. However, the Western individuals produce huge amounts of GHG emissions when compared to people living in other parts of the world. For example in 2011 the energy-related carbon dioxide emissions were 14.2 metric tons per capita in North America and 7.1 metric tons per capita in Europe whereas the world average was 4.7 metric tons per capita [2]. Therefore the reduction of GHG emission caused by individuals is important especially in the Western countries.

For climate change mitigation to be optimal, actions on both governmental level and individual level are required. The roles of the government and individuals in climate change mitigation are interrelated. A report by the Finnish Government [3] emphasizes that it is the responsibility of the policymakers to offer the infrastructure that enables the individuals to make climate-friendly choices in their everyday lives. However, in the end it is up to the individuals to actually utilize the offered infrastructure. For example, policymakers can try to enhance public transport through political decisions but the people need to actually use the public transport instead of private cars or no climate benefits are attained. Hence it is important to study the willingness of individuals to take action.

The popularity of single mitigation actions has been studied widely. More than two thirds of people report to have personally taken action to mitigate climate change in Australia and New Zealand [4], [5]. In the U.S., climate change mitigation actions most commonly taken include reducing energy consumption [6] and recycling [7]. In the UK, actions such as turning off lights when they are not in use and turning off tap while brushing teeth are popular [8]. On the whole, people are most willing to perform mitigation actions that are perceived as low-cost in terms of money, time and effort [9].

From the point of view of climate change mitigation campaigns it is not enough to know which mitigation actions are popular or what are the determinants of single actions. There is a need for information on specific groups of people engaged in similar mitigative behavior. This is because encouraging people to mitigate climate change should increasingly happen through crafted messages targeted at particular groups [10], [11]. One approach would be to address a target group on the basis of which mitigation actions (if any) the group is already engaged in [10]. To identify these groups and to decide whether targeted interventions are worthwhile, it is necessary to carry out an assessment of current behavior [12]. In order to find these groups for the purpose of an intervention, it would be helpful to know the socio-demographic characteristics of the groups. However, to our knowledge such information on groups of people engaged in similar mitigative behavior is lacking.

In order to increase the knowledge needed for targeted climate change mitigation campaigns we conducted a population-based cross-sectional study among young Finnish adults. Our aim was to identify groups of people engaged in similar climate change mitigation actions. Because there is evidence that women take voluntary action on climate change more likely than men [4], [6], we studied if there are different mitigation action groups present in the two sexes. We also studied if socio-demographic characteristics can predict which mitigation action group the individual belongs to.

## Materials and Methods

### Study population

This was a population-based cross-sectional study. The study population was the Espoo cohort established in 1991 when the cohort members were living in the city of Espoo in Helsinki metropolitan area, Finland. The cohort consists of 2568 members born between January, 1984 and March, 1990. For this 20-year follow-up the contact information of the cohort members was acquired from the Population Register Centre. A self-administered questionnaire was sent between March 2010 and June 2011 to the 2534 cohort members whose address was available. The information gathering consisted of several posting rounds as well as phone contacts. 1623 completed questionnaires were received (response rate 64.0%). The socio-economic characteristics of the study population are presented in Table 1.

**Table 1:** The socio-economic characteristics of the study population.

	Males (n = 754)	Females (n = 869)	Total (n = 1623)
**Education**	n (%)	n (%)	n (%)
Comprehensive school	45 (6.0)	60 (6.9)	105 (6.5)
Upper secondary/upper secondary and vocational school	439 (58.3)	414 (47.9)	856 (52.7)
Vocational school	122 (16.2)	123 (14.2)	245 (15.1)
Higher vocational or academic	147 (19.5)	268 (31.0)	415 (25.6)
Missing information	1	4	5
**Occupation**			
Studying	413 (56.6)	448 (53.1)	861 (54.7)
Factory/mining/construction	109 (14.9)	15 (1.8)	124 (7.9)
Office/service	158 (21.6)	296 (35.1)	454 (28.8)
Unemployed	42 (5.8)	35 (4.1)	77 (4.9)
Other (stay-at-home mother, retiree etc.)	8 (1.1)	50 (5.9)	58 (3.7)
Missing infromation	24	25	49
**Annual income (€/yr)**			
≤8400	277 (38.0)	279 (33.9)	556 (35.8)
8401–16800	241 (33.1)	300 (36.5)	541 (34.9)
≥16801	211 (28.9)	243 (29.6)	454 (29.3)
Missing information	25	47	72
**Marital status**			
Single/divorced	508 (67.5)	501 (57.9)	1009 (62.3)
Married/civil partnership/cohabitation	245 (32.5)	365 (42.1)	610 (37.7)
Missing information	1	3	4

The theme of the questionnaire was Climate change, environment and health. The questionnaire contained several sections and was partly based on questions used in the previous follow-ups and research projects [13], [14]. The study was approved by the Ethics Committee of the Oulu University Hospital District.

### Assessment of climate change mitigation actions

Engagement in climate change mitigation actions was assessed by asking: *What have you done in order to mitigate the possible climate change?* The respondents could choose any number of actions from the 16 listed actions. The listed climate change actions were modified form a national climate change communication program [15] and a report on the program published by Prime Minister’s office [16]. Two of the 16 actions were excluded from the analysis due to slight overlapping and correlation with other variables (Table S2). Respondents could also add mitigation actions not mentioned in the provided list and 4% did so. However, all of these actions had low frequencies, so none of them was included as a variable in the analysis. Hence the total number of climate change mitigation actions considered was 14 (Table 2).

**Table 2:** The frequencies of climate change mitigation actions among males and females.

Climate change mitigation action taken	Frequency (%)			P value for Χ^2^ b
	Whole study populationn = 1604^a^	Males n = 745	Females n = 859	
Recycled	1178 (73.4)	471 (63.2)	707 (82.3)	<.0001
Consumed less and produced less trash	600 (37.4)	243 (32.6)	357 (41.6)	0.0002
Used environmentally friendly products	557 (34.7)	175 (23.5)	382 (44.5)	<.0001
Cut down motoring	378 (23.6)	159 (21.3)	219 (25.5)	0.0507
Preferred public transport	957 (59.7)	347 (46.6)	610 (71.0)	<.0001
Purchased a less fuel consuming car	93 (5.8)	50 (6.7)	43 (5.0)	0.1449
Given up motoring	102 (6.4)	47 (6.3)	55 (6.4)	0.9386
Avoided flying	185 (11.5)	81 (10.9)	104 (12.1)	0.4401
Conserved energy	709 (44.2)	295 (39.6)	414 (48.2)	0.0005
Used renewable energy sources for heating	136 (8.5)	61 (8.2)	75 (8.7)	0.6969
Paid attention to the electricity consumption of home appliances	701 (43.7)	257 (34.5)	444 (51.7)	<.0001
Switched to less electricity consuming home appliances	99 (6.2)	44 (5.9)	55 (6.4)	0.6801
Demanded action from policymakers and authorities	110 (6.9)	56 (7.5)	54 (6.3)	0.3308
Participated actively in civic organizations	19 (1.2)	7 (0.9)	12 (1.4)	0.3984

a The information on the mitigation actions were missing for 19 respondents.

b Chi square test statistics (Χ^2^) was used for comparison between sexes.

### Statistical methods

The aim of this study was to identify groups of people performing similar climate change mitigation actions. We assessed if there are different mitigation action groups present among males and females. We also studied if the mitigation action group of an individual can be predicted by socio-demographic factors.

We applied latent class analysis (LCA), which is a method used to classify observations into discrete, mutually exclusive latent classes on the basis of categorical manifest variables [17]. The manifest variables of our study were the 14 mitigation actions presented in Table 2. LCA is an iterative procedure that searches for maximum likelihood parameter estimates [17]. The traditional LCA estimates two sets of parameters: class membership probabilities (gamma parameters) and item-response probabilities conditional on class membership (rho parameters, P). When covariates are added to the basic model via multinomial logistic regression, regression coefficients (beta parameters) are also estimated [18]. Because of interest in the sex differences in the climate change mitigation actions, we explored the latent class structures of males and females separately. First we identified the number of latent classes present in each of the sexes. We fit models with 2 to 6 latent classes for both sexes. 100 sets of starting values were used in order to avoid local maxima of the likelihood function. The information criteria used to assess relative model fit included Akaike information criterion (AIC), consistent Akaike information criterion (CAIC), Bayesian information criterion (BIC) and adjusted Bayesian information criterion (a-BIC). We could not use G^2^ statistic to assess absolute model fit because the reference distribution of the G^2^ statistic was not known due to the large degrees of freedom [17].

Second, we added covariates to the models in order to examine if socio-demographic variables predicted latent class membership. The covariates included education, occupation, annual income and marital status. Likelihood ratio Χ^2^ test [17] was used to test if a particular covariate was a statistically significant predictor of latent class membership.

The latent class analyses were conducted using PROC LCA version 1.2.7 developed for SAS. The procedure is provided by The Methodology Center of the Pennsylvania State University and is available online free of charge [19].

## Results

### Frequencies of the mitigation actions

The only mitigation actions performed by the majority of the study population are recycling and preferring public transport (Table 2). The actions that were significantly more often taken by females than males included recycling, consuming less and producing less trash, using environmentally friendly products, preferring public transport, conserving energy and paying attention to the electricity consumption of home appliances. None of the actions was significantly more often performed by males.

### Latent class model selection

The fit statistics of the alternative latent class models are presented in Table 3. For males, all models except the 6-class model were well identified. The CAIC and BIC suggested that the 3-class solution is optimal (Table 3). Entropy was also highest for the 3-class model, which indicates that the classification error was smallest for that model [17]. The 3-class model was also interpretable. For these reasons it was concluded that there were three latent classes present among males.

**Table 3:** Classifying of respondents according to climate change mitigation actions: the fit statistics of the alternative latent class models.

	Number of latent classes	G^2a^	Degrees of freedom	AIC^b^	CAIC^c^	BIC^d^	a-BIC^e^	Entropy	Log-likelihood
Males	2	1235.89	16354	1293.89	1456.68	1427.68	1335.59	0.66	−4218.79
	3	1102.85	16339	1190.85	**1437.84**	**1393.84**	1254.12	**0.73**	−4152.26
	4	1028.56	16324	1146.56	1477.75	1418.75	**1231.41**	0.65	−4115.12
	5	991.37	16309	**1139.37**	1554.76	1480.76	1245.78	0.70	−4096.52
	6	Not well identified							
Females	2	1201.61	16354	1259.61	**1426.53**	**1397.53**	1305.43	**0.69**	−4951.79
	3	1139.75	16339	1227.75	1481.00	1437.00	**1297.27**	0.58	−4920.86
	4	1090.97	16324	**1208.97**	1548.56	1489.56	1302.19	0.66	−4896.47
	5	Not well identified							
	6	Not well identified							

The optimum values are printed in bold.

a the likelihood ratio statistic,

b Akaike information criterion,

c consistent Akaike information criterion,

d Bayesian information criterion,

e adjusted Bayesian information criterion.

For females, models with two to four latent classes were identifiable (Table 3). The CAIC, BIC and entropy were in favor of the 2-class model. This model was interpretable, and was concluded to optimally represent the latent class structure among females.

Because the two sexes had different number of latent classes, we did not combine the sexes into one study population. Instead, the subsequent analyses were conducted separately for each sex.

### Characterization of the latent classes

The item-response probabilities (rho parameters, P) for the optimum models are presented in Table 4. On the basis of these parameters labels were assigned to the latent classes. The three latent classes of males were labeled as *the Inactive* (26%), *the Semi-active* (63%) and *the Active* (11%). The Inactive males had very low probabilities (0.00–0.27) of having engaged in any climate change mitigation actions. The action that they most likely have done is recycling (probability (P) = 0.27, 95% confidence interval (CI) 0.15–0.39). The Semi-active males have typically mitigated climate change through recycling (P = 0.73, 95% CI 0.67–0.79) and preferring public transport (P = 0.54, 95% CI 0.48–0.60). Conserving energy (P = 0.45, 95% CI 0.37–0.53), paying attention to the electricity consumption of home appliances (P = 0.39, 95% CI 0.32–0.47) as well as consuming less and producing less trash (P = 0.36, 95% CI 0.28–0.44) were also relatively popular actions among the Semi-active. It was very unlikely that the Semi-Active would have purchased less fuel consuming car (P = 0.06, 95% CI 0.04–0.09), given up motoring (P = 0.05, 95% CI 0.02–0.08), avoided flying (P = 0.09, 95% CI 0.06–0.12), used renewable energy sources for heating (P = 0.07, 95% CI 0.04–0.10), switched to less energy consuming home appliances (P = 0.06, 95% CI 0.04–0.09), demanded action from policymakers or authorities (P = 0.05, 95% CI 0.03–0.08) or participated actively in civic organizations (P = 0.00). The Active males have mitigated climate change most probably through recycling (P = 0.94, 95% CI 0.87–1.00), consuming less and producing less trash (P = 0.91, 95% CI 0.81–1.00), conserving energy (P = 0.86, 95% 0.75–0.98), paying attention to the electricity consumption of home appliances (P = 0.86, 95% CI 0.75–0.98), preferring public transport (P = 0.85, 95% CI 0.73–0.96), using environmentally friendly products (P = 0.77, 95% CI 0.66–0.89) and cutting down motoring (P = 0.54, 95% CI 0.40–0.69). A portion of this class have also demanded action from authorities (P = 0.39, 95% CI 0.25–0.52), used renewable energy sources for heating (P = 0.32, 95% CI 0.21–0.44) and avoided flying (P = 0.31, 95% CI 0.19–0.43) because of climate change. It is unlikely that even the Active males would have purchased a less fuel consuming car (P = 0.10, 95% CI 0.03–0.18) or participated actively in civic organizations (P = 0.09, 95% CI 0.02–0.16).

**Table 4:** Probability (rho parameter, P) and corresponding 95% confidence interval (CI) of having engaged in the climate change mitigation actions given the latent class membership.

Climate change mitigation action taken	Males			Females	
	The Inactive (26%)	The Semi-Active (63%)	The Active (11%)	The Semi-Active (72%)	The Active (28%)
	P (95% CI)	P (95% CI)	P (95% CI)	P (95% CI)	P (95% CI)
Recycled	0.27 (0.15–0.39)	**0.73** (0.67–0.79)	**0.94** (0.87–1.00)	**0.79** (0.75–0.82)	**0.92** (0.87–0.94)
Consumed less and produced less trash	0.01(0.00–0.03)	0.36 (0.28–0.44)	**0.91** (0.81–1.00)	0.25 (0.21–0.30)	**0.84** (0.76–0.92)
Used environmentally friendly products	0.04 (0.00–0.08)	0.23 (0.17–0.28)	**0.77**(0.66–0.89)	0.32 (0.27–0.36)	**0.77** (0.70–0.85)
Cut down motoring	0.08(0.00–0.18)	0.21 (0.17–0.26)	**0.54** (0.40–0.69)	0.19 (0.15–0.22)	0.43 (0.35–0.51)
Preferred public transport	0.12 (0.00–0.29)	**0.54** (0.48–0.60)	**0.85** (0.73–0.96)	**0.66** (0.62–0.70)	**0.85** (0.79–0.91)
Purchased less fuel consuming car	0.06 (0.02–0.10)	0.06 (0.04–0.09)	0.10 (0.03–0.18)	0.04 (0.03–0.06)	0.06 (0.03–0.10)
Given up motoring	0.03 (0.00–0.08)	0.05 (0.02–0.08)	0.22 (0.11–0.32)	0.03 (0.01–0.04)	0.16 (0.11–0.21)
Avoided flying	0.07 (0.02–0.12)	0.09 (0.06–0.12)	0.31 (0.19–0.43)	0.06 (0.03–0.08)	0.29 (0.22–0.36)
Conserved energy	0.07 (0.01–0.14)	0.45 (0.37–0.53)	**0.86** (0.75–0.98)	0.35(0.30–0.39)	**0.84** (0.76–0.91)
Used renewable energy sources for heating	0.01(0.00–0.03)	0.07 (0.04–0.10)	0.32 (0.21–0.44)	0.03 (0.02–0.05)	0.23(0.17–0.29)
Paid attention to the electricity consumption of home appliances	0.02 (0.00–0.05)	0.39 (0.32–0.47)	**0.86** (0.75–0.98)	0.42 (0.37–0.46)	**0.78** (0.71–0.85)
Switched to less electricity consuming home appliances	0.00 N.A.	0.06 (0.04–0.09)	0.18 (0.08–0.27)	0.03 (0.02–0.05)	0.15 (0.10–0.20)
Demanded action from policymakers and authorities	0.01 (0.00–0.02)	0.05 (0.03–0.08)	0.39 (0.25–0.52)	0.02 (0.01–0.03)	0.18 (0.12–0.23)
Participated actively in civic organizations	0.00 N.A.	0.00 N.A.	0.09 (0.02–0.16)	0.00 N.A.	0.05 (0.02–0.08)

Responses characterizing each latent class are in bold. N.A.  =  not available for estimation.

Among females, the two classes were labeled as *the Semi-active* (72%) and *the Active* (28%). The Semi-active females have typically recycled (P = 0.79, 95% CI 0.75–0.82) and preferred public transport (P = 0.66, 95% CI 0.62–0.70) because of climate change. Some of them have paid attention to the electricity consumption of home appliances (P = 0.42, 95% CI 0.37–0.46), conserved energy (P = 0.35, 95% CI 0.30–0.39) and used environmentally friendly products (P = 0.32, 95% CI 0.27–0.36). It is unlikely that the Semi-active females would have purchased less fuel consuming car (P = 0.04, 95% CI 0.03–0.06), given up motoring (P = 0.03, 95% CI 0.01–0.04), avoided flying (P = 0.06, 95% CI 0.03–0.08), used renewable energy sources for heating (P = 0.03, 95% CI 0.02–0.05), switched to less energy consuming home appliances (P = 0.03, 95% CI 0.02–0.05), demanded action from policymakers or authorities (P = 0.02, 95% CI 0.01–0.03) or participated actively in civic organizations (P = 0.00).The females belonging to the Active class have mitigated climate change most probably through recycling (P = 0.92, 95% CI 0.87–0.94), preferring public transport (P = 0.85, 95% CI 0.79–0.91), consuming less and producing less trash (P = 0.84, 95% CI 0.76–0.92), conserving energy (P = 0.84, 95% CI 0.76–0.91), paying attention to the electricity consumption of home appliances (P = 0.78, 95% CI 0.71–0.85) and using environmentally friendly products (P = 0.77, 95% CI 0.70–0.85). It is unlikely that the Active females would have purchased a less fuel consuming car (P = 0.06, 95% CI 0.03–0.10) or participated actively in civic organizations (P = 0.05, 95% CI 0.02–0-08). This class is hence almost identical to the Active among males, except that the Active females have less likely cut down motoring (P = 0.43, 95% CI 0.35–0.51). Interestingly no counterpart for the Inactive class of males was characterized among females.

### The socio-demographic factors predicting latent class membership

We added socio-demographic factors as covariates separately for each sex. The Active class served as a reference class in both sexes. The models with covariates are presented in Table 5.

**Table 5:** Socio-economic determinants of low climate change activity.

Covariate	Males			Females	
	The Inactive	The Semi-Active		The Semi-Active	
	OR (95% CI)	OR (95% CI)	LR test^a^ P value	OR (95% CI)	LR test^a^ P value
**Education**			0.0908		<.0001
Comprehensive school (ref)					
Upper secondary/upper secondary and vocational school	0.19 (0.03, 1.33)	0.53 (0.07, 4.02)		0.41 (0.13, 1.29)	
Vocational school	0.34 (0.04, 2.71)	0.42 (0.05, 3.73)		0.40 (0.12, 1.32)	
Higher vocational or academic	0.28 (0.04, 2.22)	0.67 (0.08, 5.67)		0.26 (0.08, 0.83)	
**Occupation**			<.0001		<.0001
Studying (ref)					
Factory/mining/construction	2.52 (0.74, 8.54)	1.67 (0.47, 5.95)		0.43 (0.10, 1.84)	
Office/service	0.77 (0.32, 1.88)	1.05 (0.44, 2.49)		0.87 (0.52, 1.46)	
Unemployed	1.94 (0.41, 9.17)	1.50 (0.31, 7.24)		0.74 (0.25, 2.17)	
Other (Stay-at-home mother, retiree etc)	0.28 (0.01, 5.52)	0.95 (0.09, 10.26)		0.40 (0.17, 0.90)	
**Annual income (€)**			<.0001		<.0001
≤8400 (ref)					
8401–16800	0.84 (0.39, 1.79)	0.39 (0.18, 0.82)		1.26 (0.79, 2.00)	
≥16801	0.92 (0.33, 2.60)	0.94 (0.35, 2.58)		1.82 (0.98, 3.39)	
**Marital status**			0.0002		0.3771
Single/divorced (ref)					
Married/civil partnership/cohabitation	0.38 (0.19, 0.77)	1.10 (0.57, 2.13)		1.21 (0.79, 1.86)	

The covariate odds ratios (OR) and 95% confidence intervals (CI) were obtained from the Latent Class Analysis and the P-values from the likelihood ratio (LR) Χ^2^ test. Note: Reference class: the Active of the corresponding sex; ^a^ Likelihood ratio test.

The men working in the factory, mining or construction branch were more likely than students to belong to the Inactive class relative to the Active class, although this result was not statistically significant (odds ratio (OR) 2.52, 95% confidence interval (CI) 0.74–8.54). In a parsimonious model (Table S1) including only covariates with p<0.05 this result was strengthened (OR 3.13, 95% CI 1.00–9.78). There was also a trend for the unemployed being less active than students but this result was not statistically significant. Income was also a predictor of latent class membership among males, but its relationship with latent class membership was not coherent. Men of the middle income group (8401–16800 €/year) were less likely to belong to the Semi-active than the Active compared to the low income group (≤8400 €/year) (OR 0.39, 95% CI 0.18–0.82). Marital status clearly predicted class membership among males. The men in a relationship (marriage, civil partnership or cohabitation) had smaller odds than single or divorced men to belong to the Inactive relative to the Active (OR 0.38, 95% CI 0.19–0.77).

Among females, holders of higher vocational or academic degree were less likely to belong to the Semi-active class than the Active class with reference to comprehensive school degree holders (OR 0.26, 95% CI 0.08–0.83). Among the occupational groups, the women belonging to the group “other” were less likely to belong to the Semi-active than to the Active compared to students (OR 0.40, 95% CI 0.17–0.90). This occupational group is diverse, and includes stay-at-home mothers, retirees and people with several occupations. Among women an increase in income tended to be associated with higher odds of belonging to the Semi-active than the Active class, but this result was not statistically significant (OR 1.82, 95% CI 0.98–3.39). In the parsimonious model (Table S1) this result was statistically significant (OR 1.90, 95% CI 1.03–3.51).

Re-estimated rho parameters obtained from the covariate models were consistent with the original interpretation and are thus not reported.

## Discussion

### Main findings

Among men we could identify three groups that differ in the extent to which they are engaged in voluntary climate change mitigation: the Inactive (26%), the Semi-active (63%) and the Active (11%). Among women, only the Semi-Active (72%) and the Active (28%) were identified. Among males, being single or divorced predicted low involvement in climate change mitigation. Among females, those without tertiary degree and those with annual income €≥16801 were less involved in climate change mitigation.

### Significance of the results for climate change mitigation campaigns

Our results provide plenty of information that can be utilized in climate change mitigation campaigns. Crafted campaigns emphasizing different mitigation actions can be targeted at the different latent classes. In addition, the information on the socio-demographics of the classes can help to find the members of a particular class. However, it should be taken into account that our study population was young and had relatively low income levels, so all the results may not be applicable in the general population.

The Active comprising 11% of males and 28% of females are already performing several mitigation actions, but even these people could do more. It was unlikely that even these most engaged individuals would have purchased a less fuel consuming car or participated in civic organizations. Maybe these actions would be worth campaigning for among the engaged, such as highly educated females or married males.

The large majority of both sexes belong to the Semi-active that recycle and prefer public transport because of climate change. Therefore it seems that the majority of people are willing to mitigate climate change but only through easy, convenient actions. These people could be the most reasonable target for climate change mitigation campaigns because they already express some interest in climate change mitigation and there are a lot of actions that they are not doing at the moment. It is important that this majority is provided with facilities that make climate change mitigation convenient (such as energy efficient appliances and affordable public transport), since the lack of enabling infrastructures is perceived as a major barrier to climate change mitigation even among people that are willing to take action [20].

It is a remarkable challenge to engage the Inactive in climate change mitigation. This group possibly includes individuals who have negative environmental views, low level of knowledge, non-environmental priorities, and a perception that their behavior does not contribute to climate change [21]. The probable diverse reasons for being Inactive should be considered carefully when climate change mitigation campaigns are planned and conducted. In this study, the most probable action the Inactive are taking is recycling (Table 4), and their capacity and willingness to perform this action could be strengthened. Campaigning for climate change mitigation could be reasonable especially among single and divorced men, who are likely to be Inactive.

Overall, the results of our study indicate that even inside a limited age range there can be great variation in the engagement with climate change mitigation. This is a challenge for climate change mitigation campaigns because it means that a single approach might not be efficient among all people.

### Validity of the results

In the latent class analysis the model selection is a critical step. The selection of the number of the latent classes is aided not only by fit statistics and parsimony but also interpretability. In our study the selection of the latent class models was straightforward since for both sexes a majority of the statistical criteria favored the same model. These models were also interpretable. Therefore the selection of the number of the latent classes is not likely to be a source of error in our study.

The mitigation actions were assessed through a questionnaire where individuals reported the actions they had taken. The possibility of information bias could not be totally eliminated because we could not ascertain the reported actions. However, assessing the taken actions by any other method would have been very difficult in practice. Also, the fact that we observed a remarkable amount on inactive individuals indicates that the study population did probably not exaggerate the mitigation actions they had taken. It is a strength of this study that we were able to assess a large variety of potential climate change mitigation actions. Because respondents could also provide information on actions not ready listed, it is not likely that we would have left outside any popular actions.

The frequencies of the mitigation actions in our study were quite well in line with those observed among the European Union citizens (aged 15 years and over) in Eurobarometer 2011 [22]. The list of actions studied and the question wordings differed to some extent but the questions that were comparable produced quite similar results. For example, 73% of our study population recycled (66% of EU citizens), 24% cut down motoring (26% of EU citizens), 12% avoided flying (9% of EU citizens) and 9% used renewable energy sources (7% of EU citizens). Therefore the probability of selection bias seems to be small and our study population seems not to consist of people especially interested in climate change mitigation. In addition, the similarity with the Eurobarometer 2011 indicates that our results could possibly be extrapolated to other European young adults. The respondents of this 20-year follow-up questionnaire were a representative sample of the baseline study population [23], which further reduces the possibility of selection bias of this study.

Making generalizations about some of our results is hard because of the characteristics of the study population. Our study population had a relatively narrow age range (20 to 27 years). Therefore we cannot make conclusions about what kind of mitigation classes there are in the whole population. In some studies older people have been found to more actively mitigate climate change [24]. Therefore there could be an even more active class identifiable among older people. However, from the viewpoint of mitigation campaigns it is especially interesting to study the mitigation actions of young adults. They are the ones whose lifestyle changes have the greatest mitigation potential in the long run. In addition, our study population included mainly people with low incomes (most of the respondents were students). In our study population less than 5% had annual income above € 33600, whereas in the whole Finnish population 33% earned € 30000 or more in 2011 [25]. Probably for this reason we could not observe a clear pattern of how income is connected to climate change mitigation actions. Therefore we cannot make generalizations about the connection between income and mitigation behavior across the whole income range of the population.

The interpretation of some of the obtained results was hard. For example, the women belonging to the group “other” were less likely to belong to the Semi-active than to the Active compared to students. The group “other” was small and diverse, so this result is challenging to interpret. Maybe these people (retirees, stay-at-home mothers) who spend more time at home have more possibilities to mitigate climate change.

One limitation of our study was the lack of information on participants’ political views. Political party support seems to be connected to pro-environmental behavior [9], [26], so it could have been a predictor of latent class membership in our study. However, the connection between political and environmental views might not be as strong in Finland as it is observed to be in other nations. This is because all the major parties in Finland include environmental protection in their agenda [27]–[30] and no party has expressed strong opposition to climate change mitigation.

### Synthesis with previous knowledge

Our results are in line with the previous finding that women more likely report to have mitigated climate change [4], [6], [26]. However, our results provided insight into why it seems that men on average are less climate-friendly. This is because among men (but not women) exists a relatively large group of inactive people that very probably have not taken any climate change mitigation actions. Presumably this group makes the whole male sex appear on average as less climate-friendly than the female sex. White (conservative) males have been reported to express denialist views about climate change more likely than other adults [31], a phenomenon known as the white male effect. It is possible that the Inactive males found in our study are an embodiment of the white male effect.

In previous studies, high education has been associated with pro-environmental behavior, also climate change mitigation [7], [24], [26]. We found this association only among females. Education likely provides a better level of knowledge about environmental issues such as climate change. However, knowledge about climate change does not always turn into actions to mitigate it [8], . Therefore there might be for example psychological, social and cultural factors that connect high education to mitigative behavior.

It was very interesting that men in a relationship (marriage, civil partnership or cohabitation) were less likely to belong to the Inactive than the Active relative to single or divorced men. This means that among the Inactive there are significantly more single men than married men or men in cohabitation. Reasons explaining this observation are unclear. Could it be that the relationship makes a man behave in a more climate friendly way? This could mean that women have influence on the behavior of their partners. It can also be that men already protecting the climate are more likely to establish a relationship and this, in turn, could indicate that environmentally conscious men are more appealing to women. To resolve the direction of the effect longitudinal studies would be needed.

The known popularity of recycling as a climate change mitigation action [7], [8] was again demonstrated in our study. However, using public transport was a surprisingly popular mitigation action in the present study. In previous studies it has been one of the least popular measures to mitigate climate change [32]. The reason for the popularity of public transport in our study is probably that our study subjects were relatively young (20 to 27 years old), had low incomes and were living mainly in an urban area where public transport is well organized and easily accessible. Our result highlights the fact that mitigation actions considered as inconvenient by some [9] may be convenient for others. This also indicates that mitigation campaigns aimed at young urban people might not need to underline the use of public transport as a way to mitigate climate change, since it is already a popular action.

## Conclusions

Our results indicate that people can be classified into clearly distinctive groups on the basis of their climate change mitigation actions by means of latent class analysis. Among both sexes the large majority has most probably mitigated climate change by recycling and preferring public transport. This is the group that could probably be encouraged to take more action. One fourth of men are very inactive when it comes to climate change mitigation: members of this group most likely have taken no action. Among both sexes there is a minority that is very active in climate change mitigation. Our study therefore illustrates to what extent young adults are engaged in climate change mitigation at the moment and hence provides valuable information for mitigation campaigns. According to our results, campaigns could be particularly targeted at single or divorced men and women without tertiary degree.

## Supporting Information

Table S1 **Socio-economic determinants of low climate change activity: the parsimonious models including only covariates with P<0.05.**
(DOC)

Table S2 **The Pearson correlation coefficients of the climate change mitigation actions.**
(DOCX)
