# Supplementary material for: Voluntary Climate Change Mitigation Actions of Young Adults: A Classification of Mitigators through Latent Class Analysis
Source: PLoS One. 2014 Jul 23;9(7):e102072. doi: 10.1371/journal.pone.0102072 (PMC4108349; doi:10.1371/journal.pone.0102072)
Supplement: Table S1 — Socio-economic determinants of low climate change activity: the parsimonious models including only covariates with P<0.05. (DOC) [file pone.0102072.s001.doc]

**Table S1.** Socio-economic determinants of low climate change activity: the parsimonious models including only covariates with P<0.05.

| **Covariate** | **Males** | | **Females** |
| --- | --- | --- | --- |
|  | The Non-Active | The Semi-Active | The Semi-Active |
|  | OR (95% CI) | OR (95% CI) | OR (95% CI) |
| **Education** |  |  |  |
| Comprehensive school (ref) |  |  |  |
| Upper secondary/ upper secondary and vocational school |  |  | 0.42 (0.14, 1.28) |
| Vocational school |  |  | 0.42 (0.13, 1.35) |
| Higher vocational or academic |  |  | 0.27 (0.09, 0.85) |
| **Occupation** |  |  |  |
| Studying (ref) |  |  |  |
| Factory/mining/construction | 3.13 (1.00, 9.78) | 1.47 (0.44, 4.84) | 0.44 (0.10, 1.88) |
| Office/service | 1.06 (0.45, 2.47) | 1.09 (0.47, 2.53) | 0.88 (0.52, 1.48) |
| Unemployed | 2.35 (0.49, 11.32) | 1.47 (0.29, 7.39) | 0.73 (0.25, 2.13) |
| Other (Stay-at-home mother, retiree etc) | 0.33 (0.01, 6.05) | 1.02 (0.10, 10.41) | 0.43 (0.19, 0.95) |
| **Income (€/yr)** |  |  |  |
| ≤8400 (ref) |  |  |  |
| 8401-16800 | 0.85 (0.41, 1.79) | 0.40 (0.19, 0.84) | 1.30 (0.82, 2.06) |
| ≥16801 | 1.00 (0.36, 2.75) | 0.93 (0.34, 2.52) | 1.90 (1.03, 3.51) |
| **Marital status** |  |  |  |
| Single/divorced (ref) |  |  |  |
| Married/ civil partnership/ cohabitation | 0.40 (0.20, 0.79) | 1.04 (0.54, 2.00) |  |

The covariate odds ratios (OR) and 95% confidence intervals (CI) were obtained from the Latent Class Analysis. Note: Reference class: the Active of the corresponding gender
