# Supplementary material for: Voluntary Climate Change Mitigation Actions of Young Adults: A Classification of Mitigators through Latent Class Analysis
Source: PLoS One. 2014 Jul 23;9(7):e102072. doi: 10.1371/journal.pone.0102072 (PMC4108349; doi:10.1371/journal.pone.0102072)
Supplement: Table S2 — The Pearson correlation coefficients of the climate change mitigation actions. (DOCX) [file pone.0102072.s002.docx]

Table S2. The Pearson correlation coefficients of the climate change mitigation actions.

|  | Recycled | Consumed less and produced less trash | Consumed more ecologically | Composted biodegradable waste | Used environmentally friendly products | Cut down motoring | Preferred public transport | Purchased a less fuel consuming car | Given up motoring | Avoided flying | Conserved energy | Used renewable energy sources for heating | Paid attention to the electricity consumption of home appliances | Switched to less electricity consuming home appliances | Demanded action from policymakers and authorities | Participated actively in civic organizations |
| --- | --- | --- | --- | --- | --- | --- | --- | --- | --- | --- | --- | --- | --- | --- | --- | --- |
| Recycled | 1.00 | 0.21 | 0.21 | 0.37 | 0.23 | 0.03 | 0.22 | -0.01 | 0.01 | 0.02 | 0.14 | 0.06 | 0.21 | 0.04 | 0.08 | 0.07 |
| Consumed less and produced less trash |  | 1.00 | 0.40 | 0.19 | 0.30 | 0.14 | 0.17 | 0.06 | 0.11 | 0.15 | 0.28 | 0.17 | 0.23 | 0.12 | 0.18 | 0.12 |
| Consumed more ecologically^a^ |  |  | 1.00 | 0.25 | 0.45 | 0.21 | 0.19 | 0.01 | 0.10 | 0.09 | 0.22 | 0.19 | 0.19 | 0.08 | 0.18 | 0.14 |
| Composted biodegradable waste^a^ |  |  |  | 1.00 | 0.22 | 0.09 | 0.15 | 0.00 | 0.09 | 0.07 | 0.10 | 0.11 | 0.16 | 0.07 | 0.05 | 0.07 |
| Used environmentally friendly products |  |  |  |  | 1.00 | 0.14 | 0.18 | 0.02 | 0.09 | 0.13 | 0.20 | 0.18 | 0.20 | 0.07 | 0.15 | 0.13 |
| Cut down motoring |  |  |  |  |  | 1.00 | 0.18 | 0.01 | 0.20 | 0.17 | 0.12 | 0.09 | 0.06 | 0.11 | 0.10 | 0.06 |
| Preferred public transport |  |  |  |  |  |  | 1.00 | -0.13 | 0.15 | 0.07 | 0.14 | 0.05 | 0.17 | 0.03 | 0.14 | 0.08 |
| Purchased a less fuel consuming car |  |  |  |  |  |  |  | 1.00 | -0.01 | 0.01 | 0.00 | 0.07 | 0.03 | 0.09 | 0.05 | 0.02 |
| Given up motoring |  |  |  |  |  |  |  |  | 1.00 | 0.15 | 0.05 | 0.07 | 0.05 | 0.05 | 0.05 | 0.09 |
| Avoided flying |  |  |  |  |  |  |  |  |  | 1.00 | 0.08 | 0.11 | 0.10 | 0.11 | 0.12 | 0.10 |
| Conserved energy |  |  |  |  |  |  |  |  |  |  | 1.00 | 0.13 | 0.31 | 0.10 | 0.13 | 0.11 |
| Used renewable energy sources for heating |  |  |  |  |  |  |  |  |  |  |  | 1.00 | 0.10 | 0.16 | 0.17 | 0.11 |
| Paid attention to the electricity consumption of home appliances |  |  |  |  |  |  |  |  |  |  |  |  | 1.00 | 0.12 | 0.14 | 0.08 |
| Switched to less electricity consuming home appliances |  |  |  |  |  |  |  |  |  |  |  |  |  | 1.00 | 0.08 | -0.00 |
| Demanded action from policymakers and authorities |  |  |  |  |  |  |  |  |  |  |  |  |  |  | 1.00 | 0.22 |
| Participated actively in civic organizations |  |  |  |  |  |  |  |  |  |  |  |  |  |  |  | 1.00 |

^a^The actions “Consumed more ecologically” and “Composted biodegradable waste” were excluded from the latent class analysis due to slight overlapping and correlation with the other variables.
